# Optic Tract Shrinkage Limits Visual Restoration After Occipital Stroke

**DOI:** 10.1161/STROKEAHA.121.034738

**Published:** 2021-07-16

**Authors:** Berkeley K. Fahrenthold, Matthew R. Cavanaugh, Subin Jang, Allison J. Murphy, Sara Ajina, Holly Bridge, Krystel R. Huxlin

**Affiliations:** Flaum Eye Institute (B.K.F., M.R.C., S.J., K.R.H.), University of Rochester, NY. Wellcome Centre for Integrative Neuroimaging, FMRIB, Nuffield Department of Clinical Neurosciences, University of Oxford, United Kingdom. Department of Neurorehabilitation and Therapy Services, The National Hospital for Neurology and Neurosurgery, Queen Square, London, United Kingdom.; Neuroscience Graduate Program (A.J.M.), University of Rochester, NY. Wellcome Centre for Integrative Neuroimaging, FMRIB, Nuffield Department of Clinical Neurosciences, University of Oxford, United Kingdom. Department of Neurorehabilitation and Therapy Services, The National Hospital for Neurology and Neurosurgery, Queen Square, London, United Kingdom.

**Keywords:** adult, magnetic resonance imaging, optic tract, retrograde degeneration, visual field

## Abstract

**Background and Purpose:**

Damage to the adult primary visual cortex (V1) causes vision loss in the contralateral visual hemifield, initiating a process of trans-synaptic retrograde degeneration. The present study examined functional implications of this process, asking if degeneration impacted the amount of visual recovery attainable from visual restoration training in chronic patients, and if restoration training impacted optic tract (OT) shrinkage.

**Methods:**

Magnetic resonance imaging was used to measure OT volumes bilaterally in 36 patients with unilateral occipital stroke. From OT volumes, we computed laterality indices (LI), estimating the stroke-induced OT shrinkage in each case. A subset of these chronic patients (n=14, 13±6 months poststroke) underwent an average of nearly 1 year of daily visual restoration training, which repeatedly stimulated vision in their blind field. The amount of visual field recovery was quantified using Humphrey perimetry, and post training magnetic resonance imaging was used to assess the impact of training on OT shrinkage.

**Results:**

OT LI was correlated with time since stroke: it was close to 0 (no measurable OT shrinkage) in subacute participants (<6 months poststroke) while chronic participants (>6 months poststroke) exhibited LI >0, but with significant variability. Visual training did not systematically alter LI, but chronic patients with baseline LI≈0 (no OT shrinkage) exhibited greater visual field recovery than those with LI>0.

**Conclusions:**

Unilateral OT shrinkage becomes detectable with magnetic resonance imaging by ≈7 months poststroke, albeit with significant interindividual variability. Although visual restoration training did not alter the amount of degeneration already sustained, OT shrinkage appeared to serve as a biomarker of the potential for training-induced visual recovery in chronic cortically blind patients.

## Abstract 2

Supplemental Digital Content is available in the text.

Damage to the primary visual cortex (V1) or its immediate afferents causes a homonymous, contra-lesional visual field defect known as cortically induced blindness (CB). Around 70% of CB occurs after occipital stroke, with trauma, surgical resection, and tumors accounting for the rest.^1^ Although partial recovery can occur spontaneously in the first few months after damage, and there is potential for training-induced vision restoration (reviewed below), CB remains a permanent condition.^1–4^

Current medical treatment for CB does not routinely include visual restoration training, instead focusing on compensatory (primarily saccade training) or substitution (primarily prism lenses) strategies.^5–8^ Nevertheless, work from our group and others has shown that visual restoration training that requires participants to detect, identify, or discriminate particular features of targets presented in the blind-field can partially restore vision in CB, as measured by both clinical perimetry and psychophysical tests of visual performance.^9–20^ Yet, these restoration studies show considerable individual variability in the efficacy of training among CB patients, and the training required tends to be intensive, laborious, and lengthy.^9–20^ As we consider introducing restoration therapies clinically, it is important to understand the root causes of this variability so as to identify which patients are most likely to benefit and when.

One factor likely to affect the potential to recover vision after V1 damage is the state of the residual visual system. Without intervention, the perimetrically defined visual deficit of patients >6 months poststroke slowly expands and worsens over time^10,18,21^—a phenomenon attributed to retrograde degeneration of early visual pathways. Studies in humans and nonhuman primates have long shown the existence of trans-synaptic retrograde degeneration in the dorsal lateral geniculate nucleus and retina after occipital lesions.^22–29^ This is thought to occur in sequence, with dorsal lateral geniculate nucleus relay cells dying first, followed by loss of retinal ganglion cells. In macaque monkeys, the magnitude of trans-synaptic retrograde degeneration varies, appearing to correlate with the size of, as well as time since the V1 lesion.^25,30^ In humans, structural magnetic resonance imaging (MRI) analyses have shown that the optic tract (OT) ipsilateral to occipital cortex damage is often reduced in size,^23,27,28,31–33^ as are the thicknesses of the retinal ganglion cells and nerve fiber layers over corresponding regions of the retina in each eye.^28,34^ Importantly, these human studies also showed considerable interindividual variability in the severity of degeneration. We now posit that since early visual neurons are responsible for conveying the bulk of sensory input to the rest of the visual system, their loss could critically limit the ability of patients with V1 damage to recover visual functions. This was recently shown to be true with respect to spontaneous recovery, which occurs with marked individual variability during the subacute period, <6 months poststroke.^34^ However, to what extent retrograde degeneration limits vision restoration induced by training interventions in the chronic (>6 months) poststroke period remains to be determined and is the primary question addressed with the present study.

## Methods

### Data Availability

De-identified data are available on FigShare: https://figshare.com/s/e86de19092d149765d42

### Participants and Study Design

The present analyses were performed on magnetic resonance imaging data from 36 CB patients (Table 1, Table I in the Data Supplement): 28 were chronic (>6 months poststroke) and 8 were subacute (<6 months poststroke). Five of the patients (all chronic) sustained hemorrhagic strokes, with the remainder sustaining ischemic strokes. Thirty-2 CB participants were recruited at the University of Rochester (United States); 4 CB patients were recruited at the University of Oxford (United Kingdom). Imaging data were also obtained from 15 visually intact controls (Table 1, Table I in the Data Supplement)—13 imaged at the University of Rochester (United States), 2 at the University of Oxford (United Kingdom). All Rochester procedures were approved by the Institutional Review Board of the University of Rochester Medical Center (No. RSRB00021951). University of Oxford procedures were conducted under ethical approval from the Oxfordshire National Health Service Research Ethics Committee (08/H0605/156) or University of Oxford (R59810/RE001). All participants gave written, informed consent, and all procedures adhered to the tenets of the Declaration of Helsinki. For each patient, we documented the nature of the stroke (ischemic or hemorrhagic), and the presence of several risk-factors, including diabetes, hypertension and migraine. Finally, we excluded patients with ocular disease (other than refractive error), neglect and neurological disease unrelated to the occipital stroke. Humphrey visual field (HVF) automated perimetry was collected in all Rochester patients save one, in one Oxford patient, and was not collected in controls.

**Table 1.**
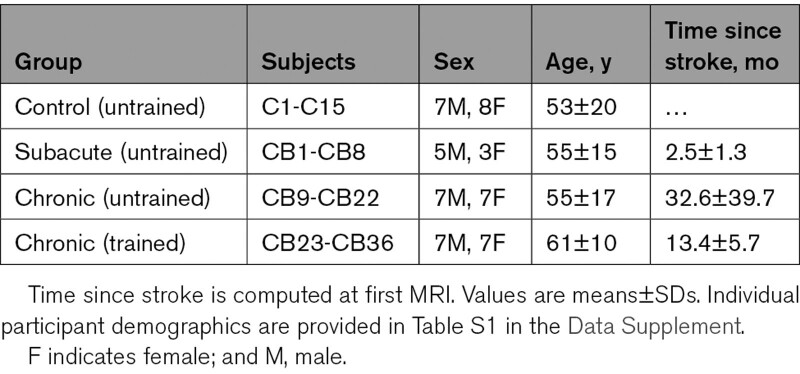
Participant Demographics

The training portion of the study was performed in a subset of chronic patients from Rochester only. Changes in HVF performance were used as the outcome measure (see Data Supplement for details of restoration training and HVF analysis protocols), which was correlated with MRI-derived OT metrics (detailed in the Data Supplement). Eligibility for the training arm of the study required that chronic CB participants meet additional criteria: (1) reliable 24–2 and 10–2 Humphrey visual field perimetry tests, with reliability defined as <20% fixation losses, false-positive and false-negative errors in either eye, (2) fixation precision better than ±1 degree relative to a fixation spot during psychophysical testing, measured using an Eyelink-1000 eye tracker (SR Research, Canada), and (3) ability to perform months of daily, in-home training, followed by a return laboratory visit in which performance on the home-training tasks was verified with fixation enforced using the Eyelink-1000 eye tracker. Only 14 of the original chronic participants met these criteria. During their return visit to the Rochester laboratory, 3 of the 14 trained patients declined (claustrophobia) or failed to meet eligibility criteria for a repeat MRI (incompatible device implantation during the training period). Thus, while we could measure training-induced changes in all 14 patients using psychophysics and HVF tests, we were unable to obtain a post-training measure of OT laterality in these 3 patients.

### MRI Procedures

For University of Rochester participants, MRI data were acquired on a 3T Magnetom Trio scanner (Siemens, Erlangen, Germany) using a 32-channel head coil. High-resolution T1-weighted anatomic volumes (MP-RAGE, magnetization prepared rapid gradient echo) were collected with a voxel size of 1 mm^3^. For University of Oxford participants, MRI data were acquired with a 3T Prisma scanner (Siemens, Erlangen, Germany) at the Oxford Centre for Functional MRI of the Brain (FMRIB). Whole-head, structural, T1-weighted scans were collected axially at a resolution of 1 mm^3^ (MP-RAGE; TR: 1900 ms; echo time: 3.97 ms; flip angle: 8°).

The OT volume analysis was adapted from prior published work.^23,32^ To account for differences in head orientation in the scanner, FMRIB software library (FSL) image analysis software (http://www.fmrib.ox.ac.uk/fsl) was used to reorient the OTs parallel to the anterior-posterior axis (*y* axis) in standard space (1 mm), and the scans were resampled parallel and perpendicular to the OTs. Details on methodology to quantify OT shrinkage are provided in the Data Supplement, and illustrated in Figure I in the Data Supplement.

### Statistical Analyses

Standard parametric tests (ie, repeated-measures ANOVAs, paired *t*-tests) were used to assess within-subject differences. For independent sample comparisons, unpaired *t*-tests were used when contrasting 2 groups; ANOVAs were used to contrast 3 or more groups. Partial eta-squared and Cohen *d* values were calculated to assess effect size for ANOVAs and *t*-tests, respectively. Linear regressions were used to model the relationship between explanatory variables and dependent outcomes, with *r* values and 95% confidence intervals (CI_95_) for rho provided, and significance estimated using a *t* test.

## Results

### OT Laterality in CB Patients

**Figure 1.**
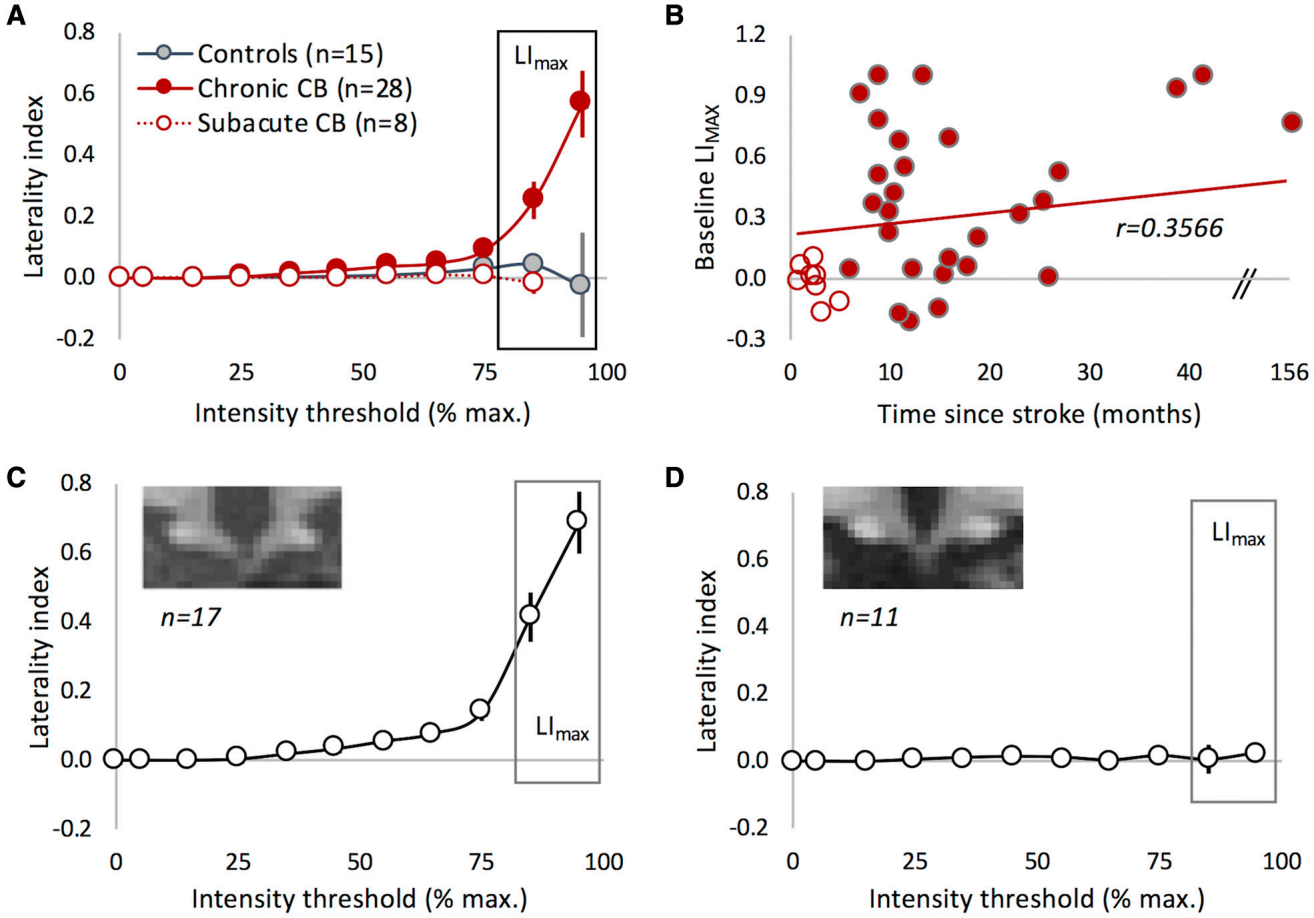
**Optic tract (OT) structure after unilateral occipital stroke.** **A**, Graph of laterality indices for different voxel intensities in cortically blind (CB) and control participants. Note the nonlateralized OTs in visually intact controls (gray symbols), and subacute CB patients (open red symbols), which contrasts with the lateralized OTs of chronic CB participants (filled red symbols). Positive LIs reflect a smaller OT ipsilateral to the damaged V1. **B**, Plot of LI_max_ vs time since stroke. Subacute patients (<6 mo poststroke): open symbols; chronic patients (≥6 mo poststroke): filled symbols. **C**, Plot of LI for different voxel intensities in the OT of lateralized CB patients. Inset: magnified T1 magnetic resonance imaging (MRI) showing smaller, right OT. **D**, Plot of LI for different voxel intensities in nonlateralized CB patients. Inset: magnified T1 MRI showing similar left and right OT sizes.

We first contrasted OT volumes in CB patients and visually intact controls, computing a laterality index (LI) for each person (Figure 1A). Control and subacute CB participants had an LI ≈0 for all OT pixel intensity thresholds (Figure 1A, gray, and open red symbols, respectively). In contrast, chronic CB participants LI values (Figure 1A, filled red symbols) became increasingly positive at higher pixel intensities. More positive LI values denoted a higher number of brighter voxels in the contralesional relative to the ipsilesional OTs. As a group, the LI_max_ (LI value for maximal OT voxel brightness) of chronic CBs was greater than in subacute CBs (t_34_=−5.27, *P*<0.0001, CI_95_=±0.073, ±0.149) or in visually intact controls (t_32_=−4.6, *P*<0.0001, CI_95_=±0.059, ±0.149). Importantly, this was not explained by chronic patients having larger visual defect sizes (area of 24-2 HVF with a pattern deviation <−5 dB, t_30_=0.47, *P*=0.641, CI_95_=±89, ±127), or severity, measured by perimetric mean deviation (PMD) averaged between the 2 eyes (t_30_=1.15, *P*=0.259, CI_95_=±1.9, ±2.6).

When considering all CB patients (subacute and chronic), time since stroke and OT laterality were positively correlated (Figure 1B: *r*=0.3566, CI_95_ for rho=0.032 to 0.613, t_42_= 2.23, *P*=0.032). LI_max_ values in subacute CB patients were close to zero (open symbols, mean±SEM=−0.016±0.08, Figure 1B), resembling control participants (LI_max_=0.043±0.02). In contrast, chronic patients had some of the highest, most positive LI_max_ values (eg, right-most solid red symbols in Figure 1B), albeit with a large range of LI_max_ values evidenced as early as 7 months poststroke.

To examine factors contributing to the heterogeneity of OT LI_max_ among chronic patients, we separated them into 2 groups: those with LI_max_>0.256 were termed lateralized (Figure 1C); those with LI_max_<0.256 nonlateralized (Figure 1D). LI_max_ of 0.256 was chosen because it is 2 SDs greater than the LI_max_ of controls. Using this classification, mean LI_max_ in lateralized chronics=0.66±0.06 (SEM), while mean LI_max_ in nonlateralized chronics=0.01±0.04.

### Chronic CB: OT Laterality Correlates With Deficit Severity and Size

**Figure 2.**
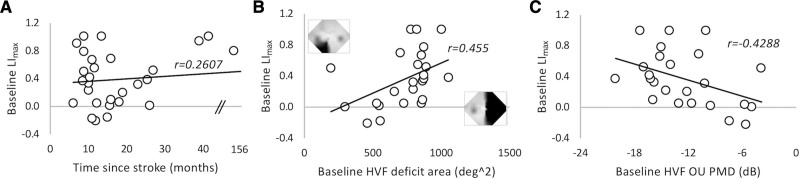
**Baseline optic tract (OT) laterality in chronic cortically blind (CB) correlates with visual defect size.** **A**, Plot of baseline LI_max_ vs time since stroke of chronic patients. **B**, Plot of baseline LI_max_ vs baseline Humphrey visual field (HVF)-defined visual deficit area. Insets show example binocular (OU) HVF composite maps at either extreme of the range, with black areas denoting visual defects. **C**, Plot of pretraining LI_max_ vs OU perimetric mean deviation (PMD) from 24-2 HVFs.

In chronic patients, LI_max_ was no longer correlated with time since stroke (Figure 2A). Instead, it was positively correlated with deficit area (Figure 2B, *r*=0.455, CI_95_ for rho=0.064 to 0.725, t_22_=2.4, *P*=0.025) and negatively correlated with PMD (Figure 2C, *r*=−0.4288, CI_95_ for rho=−0.71 to −0.03, t_22_=−2.23, *P*=0.036). Unsurprisingly, lateralized chronic patients had worse baseline PMD (−13.9±1.2 dB, t_22_=2.39, *P*=0.026, CI_95_=±2.84, ±2.23) and larger visual deficits (815±53 deg^2^, t_22_=−2.35, *P*=0.028, CI_95_=±132, ±115) than nonlateralized chronic patients (PMD: −9.98±1.3 dB, deficit area: 630±58 deg^2^). Supplementing these results, baseline visual field estimates were also derived from Goldmann kinetic perimetry (Figure IIA through IIC in the Data Supplement), which can better capture the entirety of the visual field in each eye than HVFs. LI_max_ was strongly, negatively correlated with the size of the bilateral residual visual field encompassed by the largest tested Goldmann isopter, V4e (Figure IIC in the Data Supplement, *r*=−0.6503, CI_95_ for rho=−0.84 to −0.32, t_20_=−3.83, *P*=0.001). Thus, deficit size, severity, and residual field size explained the large range of OT lateralization observed in chronic CB.

Finally, since a large volume of V1 is devoted to the representation of the central 10° vision,^35–39^ we asked if those participants whose deficit impacted this central region were more likely to be lateralized. As shown in Figure III in the Data Supplement, there was no significant correlation between 10-2 HVF PMD or area of deficit and baseline LI_max_ in our group of chronic CB patients (10-2 PMD: *r*=−0.0196, CI_95_ for rho=−0.428 to 0.395, t_21_=−0.09, *P*=0.929; 10-2 area of deficit: *r*=0.1793, CI_95_ for rho=−0.25 to 0.55, t_21_=0.84, *P*=0.410). Thus, more extensive damage to the cortical representation of the central 10° of vision did not determine OT lateralization in our cohort of chronic stroke patients.

### Chronic CB: Pretraining LI_max_ Predicts Training-Induced Visual Field Recovery

**Figure 3.**
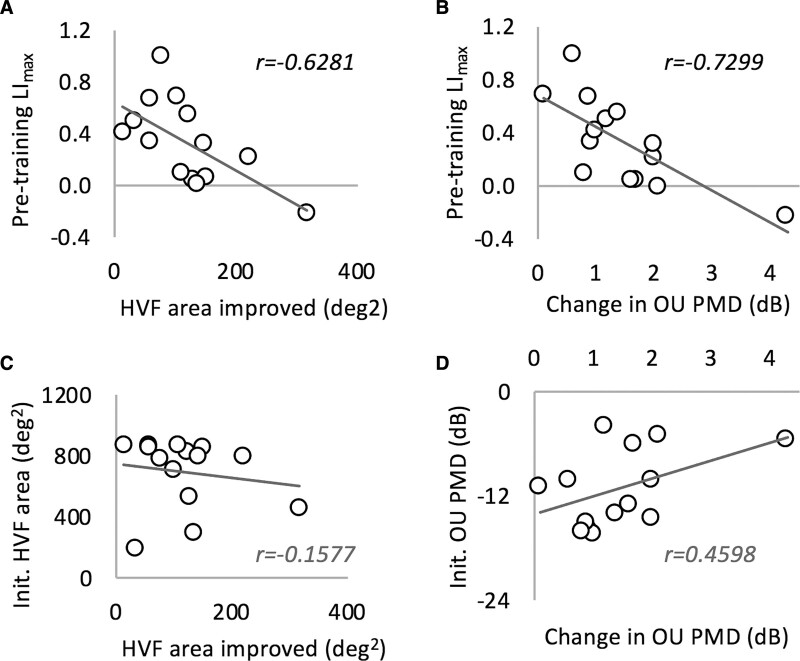
**Pretraining optic tract (OT) laterality predicts training-induced Humphrey visual field (HVF) recovery irrespective of initial deficit size.** **A**, Plot of pretraining LI_max_ vs HVF area improved by ≥6 dB. **B**, Plot of pretraining LI_max_ vs oculus uterque (OU) perimetric mean deviation (PMD) improvement attained from training. **C**, Pretraining deficit area computed from 24-2 HVFs does not correlate with area of HVF improvement post-training. **D**, Pretraining OU PMD does not correlate with change in PMD after training.

Chronic patients who trained did so on average for 364±79 (SEM) sessions of 300 trials each. At the group level, pretraining LI_max_ was inversely correlated with the area of HVF improvement ≥6 dB attained following visual discrimination training (Figure 3A: *r*=−0.6281, CI_95_ for rho=−0.87 to −0.15, t_12_=−2.8, *P*=0.016) and the PMD change (Figure 3B: *r*=−0.7299, CI_95_ for rho=−0.91 to −0.33, t_12_=−3.7, *P*=0.003). Importantly, there was no correlation between baseline deficit size and area of HVF improvement (Figure 3C: *r*=−0.1577, CI_95_ for rho=−0.64 to 0.41, t_12_=−0.55, *P*=0.592), nor between baseline oculus uterque PMD and change in PMD (Figure 3D: *r*=0.4598, CI_95_ for rho=−0.09 to 0.80, t_12_=1.79, *P*=0.099). As such, baseline deficit size/severity did not affect training-induced HVF recovery (see additional illustrations in Figure IV in the Data Supplement). Overall, our data suggest that independent of initial deficit size, and given extensive visual training in the blind field, initial structural integrity of the OTs at training onset may impact training efficacy in chronic CB patients. Given baseline variability in LI_max_, we posit that baseline LI_max_ is a key factor driving individual variability in training outcomes for this patient population.

### Chronic CB: Visual Training Does Not Appear to Change OT Laterality

Our final question was whether visual training in the chronic period altered retrograde degeneration at the level of the OTs. Our laboratory has conducted a range of training studies where measurable improvements in visual perception were elicited in chronic CB patients.^10,14,40^ Here, we analyzed the subset of 11 chronic CB patients who underwent ≈1 year of visual training, together with pre- and post-training MRI. All 11 exhibited improvements that could be verified using fixation-enforced psychophysical testing and HVF perimetry; 7 of them were lateralized at baseline (ie, before training onset), and 4 were nonlateralized.

Visual discrimination training in the blind field induced improvement ≥6 dB across an area of the HVF 101±24 deg^2^ (SEM), which translated to a 1.3±0.3 dB increase in the binocular PMD. Consistent with earlier observations that pretraining LI_max_ was negatively correlated with HVF improvement, nonlateralized chronic patients exhibited a larger area of HVF improvement than lateralized patients (Table 2), although both groups trained for a similar number of sessions (t_9_=−1.2, *P*=0.26, CI_95_=±174, ±545). PMD change, which reflects vision averaged across the entire visual field, was not sufficiently sensitive to detect this blind-field specific improvement with our present, small sample size (Table 2).

**Table 2.**
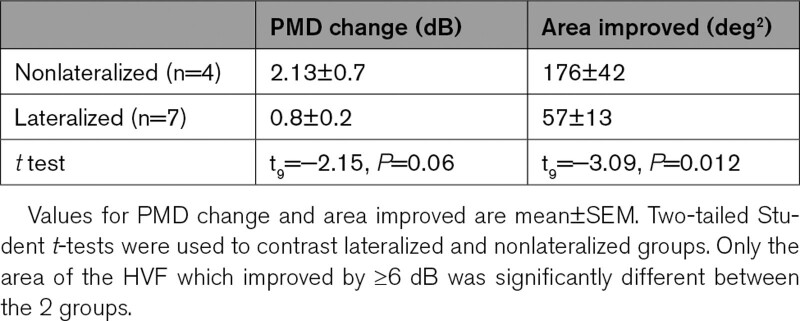
Effect of Training on HVF Perimetry in Chronic CB Patients Classified as Nonlateralized or Lateralized Based on LI_max_ Before the Onset of Training

**Figure 4.**
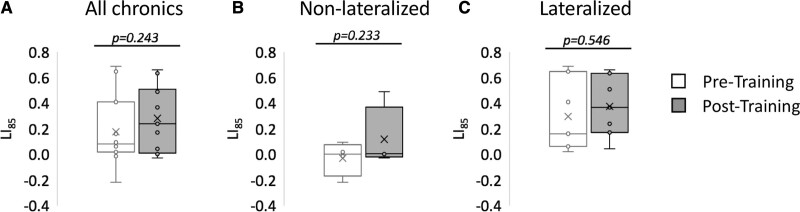
**Visual training in chronic cortically blind (CB) does not change OT laterality.** **A**, Box plot of LI_85_ across trained participants (n=11) showing no significant change as a function of training. **B**, Box plot of LI_85_ in subset of nonlateralized, chronic CB participants (n=4) before and after training. **C**, Comparable plot to A and B for lateralized, chronic CB participants (n=7).

To assess the effect of visual training on OT laterality, we analyzed LI_85_ rather than LI_max_, accounting for the fact that not every patient had voxel intensities >85% of maximum at both time-points. We found no significant difference between pre- and post-training LI_85_ across the 11 patients imaged (Figure 4A: t_10_=−1.24, *P*=0.2432, CI_95_=±0.191, ±0.177). This lack of difference persisted when data were segregated into nonlateralized (Figure 4B: t_3_=−1.49, *P*=0.233, CI_95_=±0.213, ±0.393) and lateralized cohorts (Figure 4C: t_6_=−0.64, *P*=0.5458, CI_95_=±0.263, ±0.220). Thus, visual training, which improves perception in chronic CB, may do so without significantly altering OT laterality.

## Discussion

The present study builds upon previous findings about retrograde degeneration following damage to the adult, occipital cortex, and the fundamental observation that this degeneration follows a distinct time course and pattern.^22,23,25,28,32^ We not only show strong evidence of unilateral shrinkage of the OT ipsilateral to the damaged V1 in a large cohort of chronic CB patients but also highlight the lack of measurable OT shrinkage in subacute patients. Differences in deficit size were ruled out as a possible explanation, confirming the notion^28,41^ that trans-synaptic, retrograde degeneration must require ≈6 months post-V1 stroke to be evidenced at the level of the human OTs.

### Factors Driving Heterogeneity of Chronic CB Patients

A clear observation in the present study was that chronic CB patients are not a uniform population in terms of OT degeneration, with 60% showing significant OT lateralization, but 39% having LI_max_ similar to visually intact controls (and subacute CB patients). Whether preservation of the OT - sometimes years after a stroke—in a proportion of chronic CB patients truly signifies decreased or absent retrograde degeneration remains to be determined. However, since we found no mention of this dichotomy in prior literature,^22,23,25,28,32,33,42,43^ we began to interrogate its possible causes. We ruled out time poststroke as a significant predictor of chronic LI_max_. Only one of the chronic patients in our entire sample had documented diabetes (Type 2) and they were nonlateralized. Additionally, 10/17 (59%) of the lateralized chronic patients and 7/11 (64%) of the nonlateralized chronic patients had hypertension. The incidence of migraine was similarly small across groups: 4/17 (24%) in lateralized patients and 3/11 (27%) in nonlateralized participants. All in all, in our sample, diabetes, hypertension, and migraine did not seem to be significant predictors, nor did they correlate with shrinkage of the ipsilesional OT after occipital stroke. Instead, chronic patients with larger, more severe visual deficits (measured using HVF and Goldmann perimetry) had larger LI_max_. Thus, greater damage to V1 caused increased degeneration of the ipsilesional OT, with deficit (and likely lesion) size accounting for much of the variance in chronic LI_max_. However, several other factors, which could have contributed to individual differences in retrograde degeneration after V1 damage, could not be evaluated here. We can only speculate that the relative amount of damage sustained to gray and white matter, the exact nature of treatments received in the clinic (eg, tPA), as well as genetic and epi-genetic influences on cellular repair and inflammatory responses could all influence the rate of degeneration and plastic potential of early visual pathways after occipital strokes. Nonetheless, a key question emerging was: to what extent did the degeneration sustained impact the amount of recovery these individuals could achieve with restorative interventions.

### Pretraining LI Predicts Training Efficacy in Chronic CB

A recent study^34^ reported a correlation between preserved, early visual circuitry (retinal ganglion cell layer thickness and stimulus-based early visual cortex activity) and the amount and location of spontaneous visual improvements during the subacute, poststroke period. However, over the last few decades, a major focus in the field has been to investigate visual training approaches to restore vision in chronic CB patients. These patients are of interest because they no longer exhibit spontaneous recovery,^4^ but intensive visual training can recover a range of visual abilities in the blind-field of some (but not all) these individuals (reviewed in study by Melnick et al,^15^ Matteo et al,^44^ Perez et al,^45^ and Pouget et al^46^). Such variability in the extent of recovery was clearly evidenced in the present cohort: given comparable amounts of daily training over a long period of time, chronic CB patients with nonlateralized OTs (LI_max_ close to 0) at the onset of training attained significantly larger HVF improvements than lateralized, chronic CB patients. Thus, baseline pretraining LI_max_ emerges as a biomarker able to individually predict the efficacy of visual training for recovering vision in the chronic period poststroke.

Several factors have been postulated as key for training-induced visual field recovery in chronic CB, including the preservation and re-activation by training of perilesional V1 cortex weakened, but not destroyed, by the stroke.^47^ The present findings support this hypothesis, showing that patients with the greatest preservation of retino-geniculo-striate circuits sustain the greatest perceptual benefit from visual training. This is not to say that chronic patients with greater degeneration of early circuits cannot benefit from restoration training—they can, but the amount of benefit attained for the amount of effort expended is less. Perhaps these patients’ visual recovery relies to a larger extent on extra-geniculo-striate pathways that convey information to extrastriate visual cortical areas for processing.^48–56^ Our findings would suggest that recruitment of these alternative pathways by training may be less efficient for recovering conscious vision than recruitment of residual V1.

### Visual Training May Not Change OT Laterality in Chronic CB

Finally, we asked if visual training administered in the chronic poststroke period changes LI. In patients with lateralized OTs, an interesting outcome would be to see training decrease LI, suggesting that training can reverse structural and/or functional processes associated with retrograde degeneration. For patients with nonlateralized OTs, an equally interesting outcome would be to see training prevent them from becoming lateralized, presumably by blocking further degeneration. All chronic patients who were trained attained some measure of visual recovery inside their blind field—even those who only began training a year or more after their occipital stroke (see also study by Huxlin and Cavanaugh,^10^ Cavanaugh et al,^11^ Das et al,^13^ Huxlin et al,^14^ Saionz et al^18^). However, we saw no overall change in LI_85_ as a function of training. Due to our reduced sample size, this result was clearest in lateralized patients (n=7), where visual training neither decreased nor increased OT laterality. Among the 4 nonlateralized patients, 1 converted to lateralized (suggesting that training did not prevent degeneration from progressing), but the remaining 3 did not. This is an extraordinarily difficult experiment to carry out: patients need to meet MRI eligibility twice—≈1year apart on average—in addition to performing rigorous, correctly executed visual training in their blind-field daily for ≈1 year on average. Nonetheless, additional studies using greater sample sizes are needed to sufficiently power any claims about the effect of training on the OTs in chronic patients.

### Implications for Future Work

The present findings illustrate some important structural limitations of restorative plasticity in adult, chronic stroke patients. Follow-up studies that include larger and more homogenous groups of chronic patients are needed to verify the functional implications of these results. Additionally, from a rehabilitation perspective, OT shrinkage could be measured and used to identify chronic patients who may benefit from different training strategies.

However, our results also highlight potential opportunities offered by intervening in the subacute rather than chronic poststroke period. Could training administered early as opposed to late after stroke, stop or slow the progress of retrograde degeneration? This makes intuitive sense, as retrograde degeneration in humans is well underway and clearly evidenced by structural changes relatively early in the chronic period.^25,28,34,57^ Intervening in the subacute period may catch the visual system in a state where degeneration is not yet complete. Therefore, we posit that future studies should investigate if early intervention in acute and sub-acute CB populations can slow the rate or magnitude of degeneration, keeping LI_max_ closer to zero for longer and thus, further improving the potential for training induced visual recovery once participants reach the chronic poststroke period. Even if the progression of degeneration cannot be stemmed, an intervention during the subacute period would take advantage of the greater integrity of retinal ganglion cells and the white matter tracts (including the OT) conveying information to subcortical centers to attain larger training-induced visual improvements. We recently verified this hypothesis, showing that visual training in subacute CB elicits more rapid and spatially extensive recovery than identical training in the chronic period.^18^ However, the possibility also remains that interventions started before (rather than after) retrograde degeneration is fully evidenced, may act to slow or stop its progress. If true, this would reinforce the notion that time is indeed vision after occipital strokes. More importantly, early intervention could create a larger pool of chronic patients with increased potential for further, training-induced recovery.

## Acknowledgments

Drs Fahrenthold, Bridge, and Huxlin designed the study; Drs Fahrenthold, Ajina, and Bridge collected data; Dr Fahrenthold, Dr Cavanaugh, S. Jang, A.J. Murphy, and Dr Huxlin analyzed the data; Drs Fahrenthold and Huxlin wrote the article, and all authors critically reviewed and commented on it. We thank Terrance Schaefer and Hannah Willis, who performed visual field tests, and Christine Callan for patient recruitment and her outstanding work as research coordinator, regulatory compliance monitor, and quality controller.

## Sources of Funding

This work was funded by NIH grants (R01 EY027314 and R01 EY021209 to Dr Huxlin, as well as T32 EY007125 and P30 EY001319 to the Center for Visual Science), and by an Unrestricted Grant from the Research to Prevent Blindness (RPB) Foundation to the Flaum Eye Institute. Dr Bridge was supported by a Royal Society University Research Fellowship. Dr Ajina was supported by a Wellcome Trust Clinical Research Training Fellowship and Academy of Medical Sciences Starter Grant. The Wellcome Centre for Integrative Neuroimaging is supported by core funding from the Wellcome Trust (203139/Z/16/Z).

## Disclosures

Dr Huxlin is co-inventor on US Patent No. 7,549,743. The remaining authors have no competing interests.

## Supplemental Materials

Expanded Materials and Methods

Online Table I

Online Figures I–IV

## Supplementary Material


