# Supplementary material for: Optic Tract Shrinkage Limits Visual Restoration After Occipital Stroke
Source: Stroke. 2021 Jul 16;52(11):3642–50. doi: 10.1161/STROKEAHA.121.034738 (PMC8545836; doi:10.1161/STROKEAHA.121.034738)
Supplement: Supplementary file 1 [file str-52-3642-s001.pdf]

Supplemental Material

Expanded Materials and Methods

Humphrey Visual Field (HVF) testing and analysis (n=32)

HVFs were collected monocularly in both eyes before and after training (if applicable) in all but one University of Rochester patients, and one of the Oxford patients. A Humphrey Field Analyzer II-*i* 750 was employed, using the 24-2 and 10-2 testing patterns and a white, size III stimulus (Zeiss Humphrey Systems). The 24-2 pattern included 54 locations with a sampling resolution of 6 degrees, covering the central 42 degrees of the visual field. The 10-2 pattern tested 68 locations with a sampling resolution of 2 degrees, covering the central 18 degrees of the visual field. Tests were performed with controlled fixation, using the Gaze/Blind Spot automatic settings, visual acuity corrected to 20/20, and a background luminance of 11.3 cd/m^2.

Three HVF-derived metrics were used in present analyses. First, we extracted the *Perimetric Mean Deviation (PMD)* from each 24-2 monocular test. The PMD is calculated automatically by the Humphrey STATPAC software (Zeiss Humphrey Systems) and represents the overall difference in sensitivity between the tested and expected hill of vision for an age-corrected, normal population, across the entire, central visual field. As the visual deficit in these patients is homonymous, the monocular PMD values were then averaged between the two eyes to generate a single PMD value for each patient. We then created composite, binocular HVF maps that combined the 24-2 and 10-2 patterns for each patient in Matlab as previously described [10]. In brief, luminance detection thresholds at identical test locations in monocular HVFs (both 24-2 and 10-2) were first averaged across the two eyes of each patient before natural-neighbor interpolation was applied between test locations with 0.1 deg^2 resolution. The resulting composite visual fields covered a total area 1,616 deg^2 in size. Difference maps were generated between HVFs collected pre- and post-training by subtracting the initial, composite, non-interpolated HVF from the second HFV, then interpolating the difference to create a smooth map of visual sensitivity change. From these difference maps, we calculated two additional metrics of change in the visual field: (1) *the area of visual deficit*, encompassing a region defined by pattern deviation less than -5dB; and (2) the *area of visual field where sensitivity improved by ≥6dB,* a value selected because it was double the HVF test/retest variability (Humphrey STATPAC, Zeiss Humphrey Systems).

Visual discrimination training in the blind field (n=14)

Fourteen of the chronic CB participants from the Rochester site, who met additional criteria detailed in the Methods section of the manuscript, underwent visual restoration training, with detailed behavioral outcomes published as part of prior studies [10,14,40].

In brief, prior to home-training, performance on a range of psychophysical tasks was collected in the laboratory with eye-tracker-enforced fixation control. Eye position was tracked binocularly using an Eyelink 1000 eye tracker (SR Research, Mississauga, Ontario, Canada) and custom software programmed in MATLAB (The MathWorks, Natick, MA) with Psychtoolbox [58]. During each trial, subjects were asked to fixate a small target at the center of a luminance-

calibrated CRT monitor (HP 7217A, 48.5x31.5 cm, 1024x640 pixel resolution, 120 Hz frame rate). If this was done successfully, stimuli 5˚ in diameter were presented in either intact or blind regions of the visual field, with the viewing distance to the CRT monitor enforced by a chin/forehead rest. We allowed our subjects a fixation window of only ±1° around the fixation spot. If the eyes deviated from this window prior to, or during stimulus presentation, the trial was aborted, reshuffled and added onto the remaining trials. Two blind-field training locations were selected in each participant, on independent y-axes, at least 5° apart. At these locations, subjects were asked to discriminate either the left- or rightward direction of motion of random dot stimuli (denoted "*Motion*" in **Supplemental Table I**) or the orientation (vertical or horizontal) of static, non-flickering Gabor patches (denoted "Orientation" in **Supplemental Table I**). Eight of the patients (~half the trained participants) underwent *motion* training at one blind field location and *orientation* training at a second, non-overlapping location. The rest were trained identically at two separate locations, either on motion or orientation discrimination. Training was started at the first blind-field location on a given y-axis, where discrimination performance on the selected task dropped from above-chance to chance following a 1° lateral movement further into the blind region of the visual field.

After completing baseline testing in-lab, CB participants were sent home to train on their personal home computers with a lab-issued chin/forehead rest and Matlab training program. They were asked to perform 300 trials per day, per blind-field location, at least five days per week, for several months. Home training was performed without an eye tracker, but participants were regularly reminded to fixate precisely. Weekly analysis of data log files automatically generated by the training software and emailed to the lab was used to track home progress. Once performance stabilized at levels approaching those at equivalent locations in the intact field of vision (measured during pre-tests), the training location was moved 1° deeper into the blind field along the x-axis. Participants had to demonstrate recovery in at least one trained location or have completed at least 6 months of training to return to the laboratory for verification of home-training performance. This involved repeating baseline, psychophysical tests using eye-tracker enforced fixation during stimulus presentation. Only if at-home performance was replicated under these conditions, was the participant's data included in the present analyses.

We previously showed that the amount of HVF improvement attained by chronic CB patients is independent of the type of visual discrimination training (motion or orientation) administered to the blind field [10]. The 14 chronic patients included here trained an average (± SEM) of 364±79 sessions (i.e., nearly 1 year) of 300 trials each, on their respective tasks.

<u>*Optic Tract Analysis*</u>*:* Our optic tract volume analysis was adapted from that used by Millington and colleagues [32] and Bridge and colleagues [23]. To account for differences in head orientation in the scanner, FMRIB software library (FSL) image analysis software (http://www.fmrib.ox.ac.uk/fsl) was used to reorient the optic tract to be parallel to the anterior-posterior axis (y-axis) in standard space (1mm), and the scans were resampled parallel and perpendicular to the optic tract. Mirrored masks of equal size were hand-drawn over the two optic tracts in each brain slice of a given subject, starting three slices posterior to the beginning of the optic chiasm and continuing posteriorly until the optic tracts were no longer distinct from surrounding structures (**Supplemental Figure I**).

Within each subject, the volume of each optic tract was then calculated from these masks as follows: first, we established the maximum voxel intensity (range from 0 to 255) across the two optics tracts. We then counted the number of voxels within each OT's masks that had brightness values between 5 and 95% of this maximal brightness. From this basic analysis, we computed the *Laterality Index (LI)* as a means of quantifying the relative difference in estimated volume between the two optic tracts of each participant.

For V1-stroke patients,

$$\mathrm{LI} = \frac{(\mathrm{cOT} - \mathrm{iOT})}{(\mathrm{cOT} + \mathrm{iOT})}$$

where cOT = number of voxels with brightness 5-95% of maximum in the contralesional OT
iOT = number of voxels with brightness 5-95% of maximum in the ipsilesional OT

For visually-intact, control participants,

$$\mathrm{LI} = \frac{(\mathrm{rOT} - \mathrm{lOT})}{(\mathrm{rOT} + \mathrm{lOT})}$$

Where rOT = number of voxels with brightness 5-95% of maximum in the right OT
lOT = number of voxels with brightness 5-95% of maximum in the left OT

Laterality indices for brightness levels up to a certain maximum were denoted $LI_{max\ brightness\%}$ - for instance, the laterality index for voxels 5-85% of maximum brightness was denoted LI_{85}. All subjects in the present study had values for either LI_{95}, LI_{85} or both, but some patients lacked pixels with intensity thresholds >85%. As such, unless indicated otherwise, we denoted participants' LI as LI_{max}, allowing us to compute their laterality index, no matter their individual maximal pixel intensity.

Supplemental Table I. Participant demographics. F, female; M, male; MRI, magnetic resonance imaging; HVF, Humphrey visual field. N/A: not applicable. Time since stroke is computed at first MRI.

Subjects	Sex	Age (yrs)	Time since stroke (mths)	Post-training MRI	Impaired hemifield	Group	Baseline HVF	Training
**C1**	M	30	N/A	N/A	N/A	Control	N/A	N/A
**C2**	F	31	N/A	N/A	N/A	Control	N/A	N/A
**C3**	F	27	N/A	N/A	N/A	Control	N/A	N/A
**C4**	M	23	N/A	N/A	N/A	Control	N/A	N/A
**C5**	F	64	N/A	N/A	N/A	Control	N/A	N/A
**C6**	F	43	N/A	N/A	N/A	Control	N/A	N/A
**C7**	M	25	N/A	N/A	N/A	Control	N/A	N/A
**C8**	F	61	N/A	N/A	N/A	Control	N/A	N/A
**C9**	M	71	N/A	N/A	N/A	Control	N/A	N/A
**C10**	M	76	N/A	N/A	N/A	Control	N/A	N/A
**C11**	F	80	N/A	N/A	N/A	Control	N/A	N/A
**C12**	F	59	N/A	N/A	N/A	Control	N/A	N/A
**C13**	F	65	N/A	N/A	N/A	Control	N/A	N/A
**C14**	M	69	N/A	N/A	N/A	Control	N/A	N/A
**C15**	M	66	N/A	N/A	N/A	Control	N/A	N/A
**CB1**	M	68	0.7	N/A	Left	Subacute	Yes	No
**CB2**	F	42	1.2	N/A	Right	Subacute	Yes	No
**CB3**	M	67	2.4	N/A	Left	Subacute	Yes	No
**CB4**	M	42	2.7	N/A	Right	Subacute	Yes	No
**CB5**	F	61	2.7	N/A	Left	Subacute	Yes	No
**CB6**	M	61	3.3	N/A	Right	Subacute	Yes	No
**CB7**	F	29	5.0	N/A	Left	Subacute	Yes	No
**CB8**	M	68	2.2	N/A	Left	Subacute	Yes	No
**CB9**	F	42	7.0	N/A	Right	Chronic	No	No
**CB10**	F	69	8.5	N/A	Right	Chronic	Yes	No
**CB11**	F	75	9.0	N/A	Right	Chronic	Yes	No
**CB12**	M	57	11.0	N/A	Left	Chronic	Yes	No
**CB13**	M	54	12..0	N/A	Left	Chronic	Yes	No
**CB14**	M	68	13.5	N/A	Left	Chronic	Yes	No
**CB15**	M	53	51.0	N/A	Left	Chronic	No	No
**CB16**	F	34	25.5	N/A	Right	Chronic	Yes	No
**CB17**	F	55	39.0	N/A	Right	Chronic	No	No
**CB18**	M	55	41.5	N/A	Right	Chronic	Yes	No
**CB19**	M	30	156.0	N/A	Right	Chronic	No	No
**CB20**	M	79	19.0	N/A	Right	Chronic	Yes	No
**CB21**	F	75	15.5	N/A	Left	Chronic	Yes	No
**CB22**	F	28	27.0	N/A	Right	Chronic	Yes	No
**CB23**	M	58	6.0	No	Right	Chronic	Yes	Motion
**CB24**	M	63	9.0	Yes	Right	Chronic	Yes	Motion/Ori
**CB25**	F	36	9.0	Yes	Right	Chronic	Yes	Motion
**CB26**	F	63	10.0	Yes	Right	Chronic	Yes	Motion
**CB27**	M	54	10.0	No	Right	Chronic	Yes	Motion
**CB28**	F	52	10.5	Yes	Left	Chronic	Yes	Motion/Ori
**CB29**	M	72	11.0	Yes	Right	Chronic	Yes	Motion/Ori
**CB30**	M	67	11.5	Yes	Right	Chronic	Yes	Motion/Ori

**CB31**	F	59	12.0	Yes	Left	Chronic	Yes	Orientation
**CB32**	M	62	16.0	Yes	Right	Chronic	Yes	Motion/Ori
**CB33**	M	77	16.0	Yes	Right	Chronic	Yes	Motion/Ori
**CB34**	F	68	18.0	Yes	Left	Chronic	Yes	Motion
**CB35**	F	59	23.0	No	Left	Chronic	Yes	Motion/Ori
**CB36**	F	68	26.0	Yes	Left	Chronic	Yes	Motion/Ori

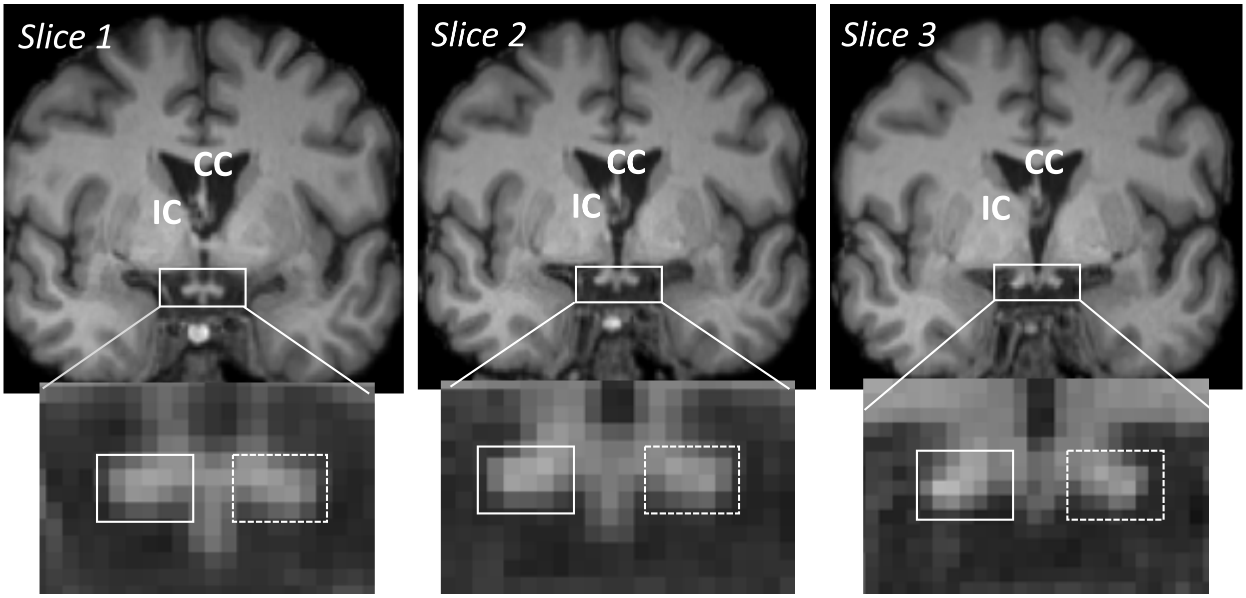

Supplemental Figure I. Optic tract analysis. Consecutive brain slices (example shown is from patient CB18) were obtained starting three slices posterior to the beginning of the optic chiasm. Mirrored masks of equal size were drawn over the two optic tracts in FSLEyes (solid and dashed boxes in magnified insets below each brain slice). Solid boxes: contralesional optic tract, dashed boxes: ipsilesional optic tract. IC = internal capsule, CC = corpus callosum.

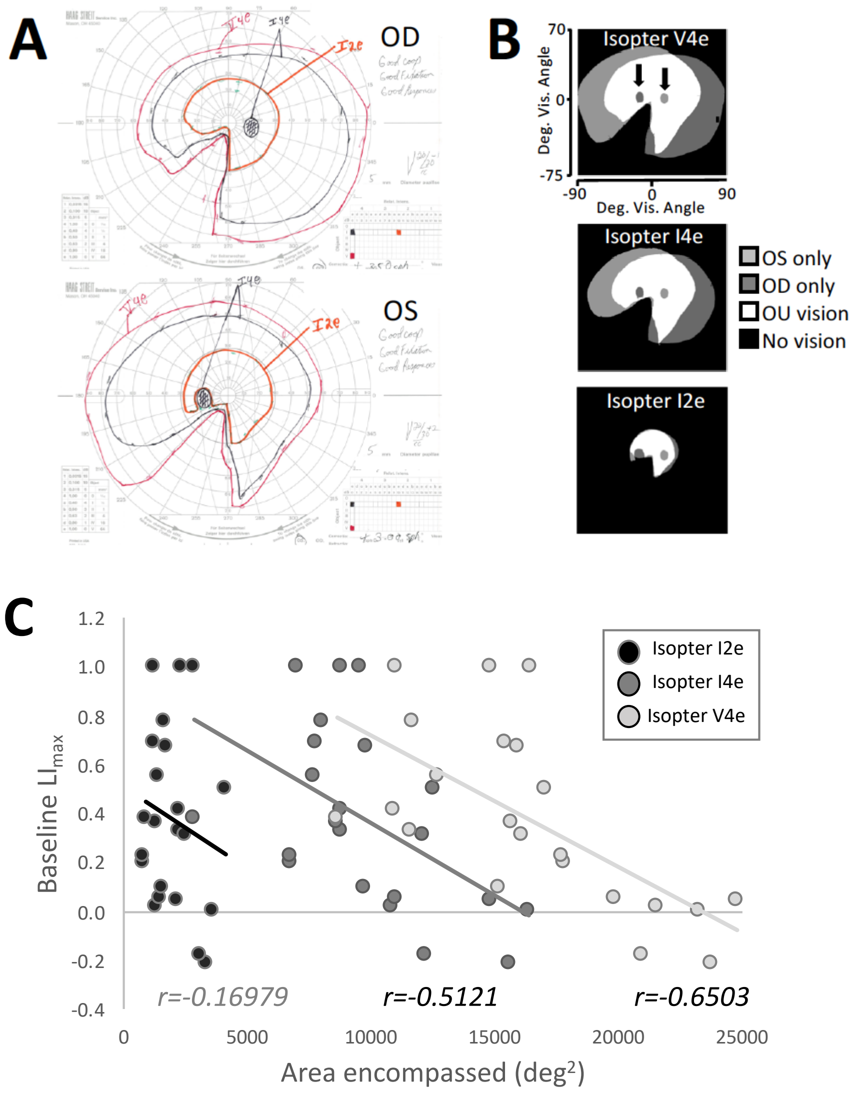

Supplemental Figure II. In chronic CB, the size of visual field captured by Goldmann perimetry correlates with baseline LI_{max}. A. Goldmann kinetic perimetry was performed monocularly on 22 of the chronic CB patients recruited at the University of Rochester Flaum Eye Institute (not all patients underwent both Humphrey and Goldmann perimetry). During Goldmann testing, patients were presented with a series of single light stimuli of a specific size and intensity, which the technician moved in and out of their visual field: the I2e stimulus (isopter 1) had a brightness of 1 asb, and covered an area of 0.25 mm^2; the I4e stimulus (isopter 2) was identical in size as I2e, but had a brightness of 10 asb; the V4e stimulus (isopter 3) had a brightness of 1000 asb, and a stimulus area of 64 mm^2.The patient signaled detection of each light via a button press, which the technician recorded, using them to hand-draw approximate fields of vision for each measured isopter. Each monocular Goldmann field was combined with that from the other eye and the area of the visual field encompassed by each isopter was analyzed in MatLab to quantify the area of vision inside each isopter. **B.** Goldmann visual fields were first aligned to a blank, reference Goldmann test form, and masks of each isopter were created by hand-tracing the field boundary on a computer. OD and OS masks were combined to create an OU masks for each

isopter. The sum of the pixels per OU mask was then calculated and converted to degrees of visual angle to determine the relative area of vision as measured by each isopter. Black arrows in top figure indicate the anatomical blind spots. **C.** Plot of Pre-training LI_{max} *versus* baseline Goldmann field of vision area encompassed by isopters I2e, I4e, and V4e. There was no significant correlation between LI_{max} and the smallest, dimmest isopter I2e (grey r value). A significant, negative correlation was only noted between LI_{max} and the two brightest isopters, denoted with black r values (I4e: t_{20}=-2.67, C_{95} *for rho= -0.767 to -0.116, p=0.015,* and V4e: t_{20}=-3.83, C_{95} *for rho= -0.841 to -0.316, p=0.001*). V4e encompassed the largest (and thus most accurate) estimate of the residual visual field in our sample of patients.

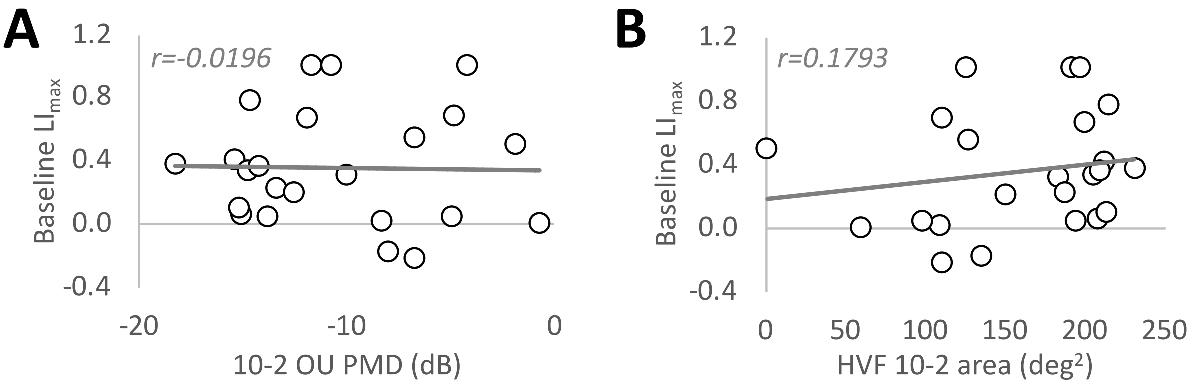

Supplemental Figure III. Pre-training LI_{max} is not impacted by the amount of deficit occupying the central 10˚ of the visual field in chronic CB. A. Plot of baseline LI_{max} *versus* baseline, binocular (OU) PMD averaged from monocular (left and right eyes) HVF 10-2 and illustrating a total lack of significant correlation (grey r value, t_{21}*=-0.09, CI_{95} for rho=-0.428 to 0.395, p=0.929*). **B.** Plot of pre-training LI_{max} *versus* baseline, binocular deficit area computed from monocular, HVF 10-2, also showing lack of significant correlation (grey r value, t_{21}*=0.84, CI_{95} for rho=-0.251 to 0.55, p=0.410*).

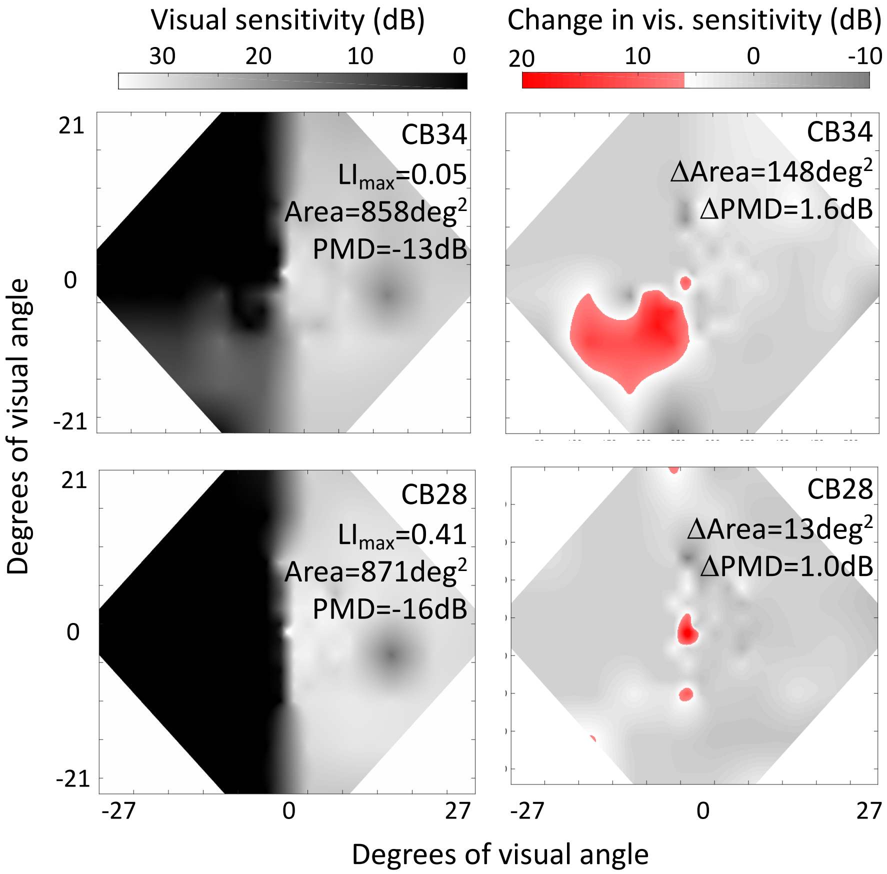

Supplemental Figure IV. Pre-training LI_{max} is the best predictor of training-induced HVF improvement. Example composite pre-training maps (left column) and change maps (right column) of 2 chronic CB patients with large, initial HVF deficits but dramatically different LI_{max}. As indicated in the main text and Fig. 3, initial deficit area and severity (PMD) did not predict visual improvement - initial LI_{max} did. See text for descriptive statistics.
