# Supplementary material for: Optic Tract Shrinkage Limits Visual Restoration After Occipital Stroke
Source: Stroke. 2021 Jul 16;52(11):3642–50. doi: 10.1161/STROKEAHA.121.034738 (PMC8545836; doi:10.1161/STROKEAHA.121.034738)
Supplement: Supplementary file 2 [file str-52-3642-s002.pdf]

American Heart Association | American Stroke Association®

Acknowledgment Permission Form

Journal Stroke
Manuscript Number STROKE/2021/034738
First Author Berkeley Fahrenthold
Title of Work Optic tract shrinkage limits visual restoration after occipital stroke

Authors must provide written permission/approval from all individuals mentioned by name in the Acknowledgments section of a submitted manuscript. By signing this form, any and all acknowledged persons therefore state that they have read and approved the mention of their names in the Acknowledgment section of the aforementioned paper.

Name (1)	Terrance Schaefer	Signature: Terrance Schaefer — Digitally signed by Terrance Schaefer Date: 2020.11.17 11:47:14 -05'00'	Date 11/17/202[illegible]
Name (2)	Christine Callan	Signature: Christine Callan — Digitally signed by Christine Callan Date: 2020.11.17 09:16:20 -05'00'	Date 11/17/202[illegible]
Name (3)	Hannah Willis	Signature: [illegible]	Date 5/12/2021
Name (4)		Signature	Date
Name (5)		Signature	Date
Name (6)		Signature	Date
Name (7)		Signature	Date
Name (8)		Signature	Date
Name (9)		Signature	Date
Name (10)		Signature	Date
Name (11)		Signature	Date
Name (12)		Signature	Date
Name (13)		Signature	Date
Name (14)		Signature	Date
Name (15)		Signature	Date
Name (16)		Signature	Date
Name (17)		Signature	Date
Name (18)		Signature	Date
Name (19)		Signature	Date
Name (20)		Signature	Date
