# Supplementary material for: Optic Tract Shrinkage Limits Visual Restoration After Occipital Stroke
Source: Stroke. 2021 Jul 16;52(11):3642–50. doi: 10.1161/STROKEAHA.121.034738 (PMC8545836; doi:10.1161/STROKEAHA.121.034738)
Supplement: Supplementary file 3 [file str-52-3642-s003.pdf]

| | |
|---|---|
| **From:** | Sacco, Ralph L |
| **To:** | Erinn Cain |
| **Subject:** | Re: [EXTERNAL] FW: [EXT] STROKE/2021/034738D-AR1: Request Final Files |
| **Date:** | Friday, May 14, 2021 8:59:57 AM |
| **Attachments:** | image001.png |

*** **CAUTION:** This email originated from outside of the **American Heart Association**. Do **not** click links or open attachments unless you recognize the sender and know the content is safe. ***

I will allw the late addition.  Thanks.

Ralph

Ralph L. Sacco MD MS FAHA FAAN
Professor and Olemberg Chair of Neurology
Executive Director McKnight Brain Institute
Chief of Neurology Jackson Memorial Hospital
Director, UM Clinical & Translational Science Institute
Senior Associate Dean for Clinical & Translational Science
Miller School of Medicine, University of Miami
Past President, American Academy of Neurology 2019-21
Office: 305-243-7519

*The information contained in this transmission may contain privileged and confidential information, including patient information protected by federal and state privacy laws. It is intended only for the use of the person(s) named above. If you are not the intended recipient, you are hereby notified that any review, dissemination, distribution or duplication of this communication is strictly prohibited. If you are not the intended recipient, please contact the sender by reply email and destroy all copies of the original message.*

On May 13, 2021, at 11:37 AM, Erinn Cain <Erinn.Cain@heart.org> wrote:

**CAUTION:** This email originated from outside the organization. **DO NOT CLICK ON LINKS** or **OPEN ATTACHMENTS** unless you know and trust the sender.

Dear Ralph,

The authors of this accepted manuscript have requested that an author be added. Please see their note below. It is journal policy not to allow authorship changes post-acceptance; would you like to make an exception?

I have attached the accepted manuscript PDF for your reference.

Thank you,

Erinn

<image001.png> | Erinn Cain
Assistant Managing Editor, Stroke
American Heart Association
200 5th Avenue | Waltham | MA | 02451
O 781.902.4412

**Explore the [Abstracts From the American Stroke Association 2021 International Stroke Conference]**

---

**From:** Huxlin, Krystel <khuxlin@UR.Rochester.edu>
**Sent:** Wednesday, May 12, 2021 9:15 AM
**To:** Erinn Cain <Erinn.Cain@heart.org>
**Cc:** Sara Ajina <sara.ajina@oriel.ox.ac.uk>; Holly Bridge <holly.bridge@ndcn.ox.ac.uk>; Fahrenthold, Berkeley <Berkeley_Fahrenthold@URMC.Rochester.edu>
**Subject:** Re: [EXT] STROKE/2021/034738D-AR1: Request Final Files

*** **CAUTION:** This email originated from outside of the **American Heart Association**. Do **not** click links or open attachments unless you recognize the sender and know the content is safe. ***

Dear Erinn,

Thank you for reaching out. We are checking and triple checking everything and in doing so, we have come to the conclusion that we need to add Dr. Sara Ajina PhD to the author list. While we realize that it is unusual for an author to be added at this time, the exclusion of Dr. Ajina was an oversight by the study team. She contributed both to data collection and intellectually to the early development of the study. To confirm that this is not a frivolous statement, she appeared on an abstract describing aspects of this work 2 years ago:

Fahrenthold, B.K., Cavanaugh, M.R., Murphy, A., **Ajina, S**., Sahraie, A., Bridge, H. and Huxlin, K.R. (2019) Effect of Visual Training on Optic Tract Degeneration after V1 Lesions. OSA FVM, Washington DC. (talk). Journal of Vision 19 (15), 35-35.

We believe that our initial omission of Dr. Ajina from the author list on our manuscript would not be a fair reflection of her contributions to this work. She has read, edited and approved the latest version of the manuscript and is CCed on this communication with you. As such, we wanted to let you know that - with your permission - the final clean version that will be uploaded to the web site will include the addition of Dr. Ajina to the author list, and will detail her specific contributions in the appropriate section of the manuscript (Acknowledgements).

We apologize for our erroneous omission of this co-author and respectfully request that we be allowed to include her in the published manuscript.

Sincerely,

Krystel Huxlin (on behalf of co-authors)

_______________________________________________

James V. Aquavella Professor of Ophthalmology
Associate Chair for Research, David & Ilene Flaum Eye Institute
Associate Director, Center for Visual Science
URMC Ombudsperson
University of Rochester
601 Elmwood Ave Box 314
Rochester, NY 14642 USA
Ph (585) 275-5495

On May 12, 2021, at 8:32 AM, erinn.cain@heart.org wrote:

May 12, 2021

STROKE/2021/034738D-AR1

Dear Prof. Huxlin,

Thank you for your email, and for providing the Acknowledgment form. Final files are needed for your manuscript "Optic tract shrinkage limits visual restoration after occipital stroke".

Please refer to your decision letter for the specific items needed. The files are needed before your manuscript can enter the production cycle.

Please click the link below to upload your best and final files. Before uploading files, please read the upload instructions carefully.

https://stroke-submit.aha-journals.org/cgi-bin/main.plex?el=A7Hn6Cuyt3B2EkmG5BS3A9ftd7ZAyOxrOtmscLIG5a94iswZ

If you have any questions, feel free to contact me at erinn.cain@heart.org.

Sincerely,

Erinn Cain
Assistant Managing Editor
Stroke

<408245_1_merged_1620822707.pdf>
