# Supplementary material for: Optic Tract Shrinkage Limits Visual Restoration After Occipital Stroke
Source: Stroke. 2021 Jul 16;52(11):3642–50. doi: 10.1161/STROKEAHA.121.034738 (PMC8545836; doi:10.1161/STROKEAHA.121.034738)
Supplement: Supplementary file 4 [file str-52-3642-s004.pdf]

Change of Authorship Form

(Must be completed and signed by ALL authors)

Please check all that apply

✔ New author(s) have been added (in addition to this form, all new authors must complete the copyright transfer agreement and conflict of interest disclosure.

____ Change in order of authorship.

____ An author wishes to remove his/her name. An author's name may only be removed his/her own request and a letter signed by the author should accompany this form

Manuscript Number STROKE/2021/034738D-AR1

Manuscript Title Optic tract shrinkage limits visual restoration after occipital stroke

Former Authorship

Please list ALL AUTHORS in the same order as the original submission. For more than 12, use an extra sheet.

Print Name		Print Name	
Name (1)	Berkeley Fahrenthold	Name (7)	
Name (2)	Matthew Cavanaugh	Name (8)	
Name (3)	Subin Jang	Name (9)	
Name (4)	Allison J. Murphy	Name (10)	
Name (5)	Holly Bridge	Name (11)	
Name (6)	Krystel R. Huxlin	Name (12)	

New Authorship

All authors must sign below agreeing to the changes in authorship. The authorship order must reflect the authorship order of the manuscript.

	Name	Signature	Date
Name (1)	Berkeley Fahrenthold	Berkeley Fahrenthold Digitally signed by Berkeley Fahrenthold Date: 2021.05.17 14:08:18 -04'00'	5/17/2021
Name (2)	Matthew Cavanaugh	Cavanaugh, Matthew Digitally signed by Cavanaugh, Matthew Date: 2021.05.17 14:37:35 -04'00'	5/17/2021
Name (3)	Subin Jang	[illegible]	5/18/2021
Name (4)	Allison J. Murphy	Allison J. Murphy Digitally signed by Allison J. Murphy DN: cn=Allison J. Murphy, o, ou, email=allison_murphy@urmc.rochester.edu, c=US Date: 2021.05.17 19:35:33 -04'00'	5/17/2021
Name (5)	Sara Ajina	[illegible]	5/18/2021
Name (6)	Holly Bridge	[illegible]	5/17/2021
Name (7)	Krystel R. Huxlin	Krystel Huxlin Digitally signed by Krystel Huxlin DN: cn=Krystel Huxlin, o=University of Rochester, ou=Flaum Eye Institute, email=khuxlin@ur.rochester.edu, c=US Date: 2021.05.17 13:14:21 -04'00'	5/17/2021
Name (8)			
Name (9)			
Name (10)			
Name (11)			
Name (12)			

Please scan and email to stroke@strokeahajournal.org.
