# Supplementary figures and images for: Optic Tract Shrinkage Limits Visual Restoration After Occipital Stroke
Source: Stroke. 2021 Jul 16;52(11):3642–50. doi: 10.1161/STROKEAHA.121.034738 (PMC8545836; doi:10.1161/STROKEAHA.121.034738)

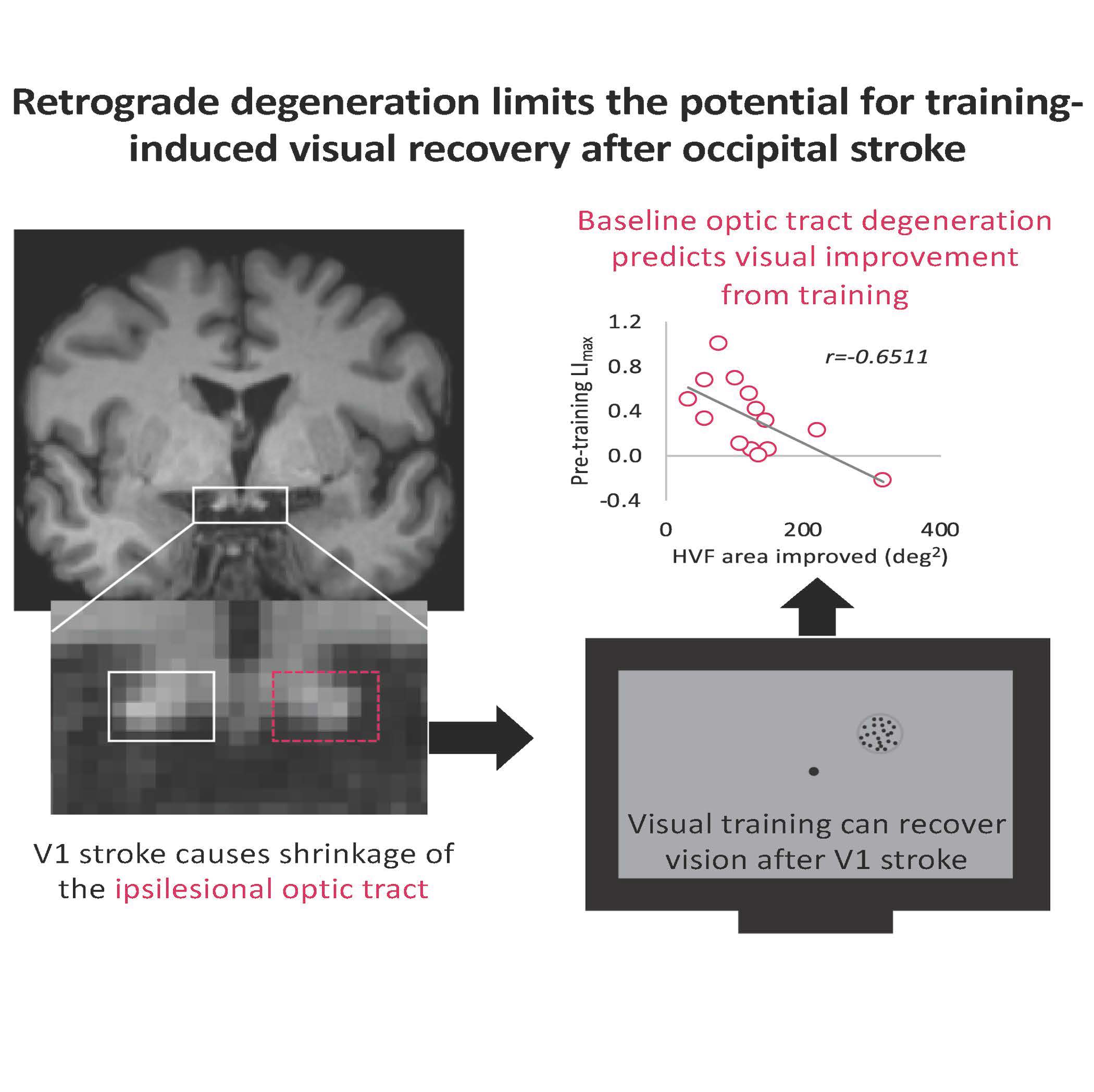

Supplement: Supplementary file 5 [file str-52-3642-s005.jpg]
